# Disability Is Not a Burden: The Relationship between Early Childhood Disability and Maternal Health Depends on Family Socioeconomic Status

**DOI:** 10.1177/00221465231167560

**Published:** 2023-04-25

**Authors:** Laurin E. Bixby

**Affiliations:** 1 University of Pennsylvania, Philadelphia, PA, USA

**Keywords:** ableism, disability, family, health, poverty

## Abstract

Narratives rooted in ableism portray disabled children as burdens on their families. Prior research highlights health disparities between mothers of disabled children and mothers of nondisabled children, but little is known about how socio-structural contexts shape these inequities. Using longitudinal data from the Future of Families and Child Wellbeing Study (n = 2,338), this study assesses whether the relationship between early childhood disability and maternal health varies by household socioeconomic status (SES). Findings reveal that, on average, mothers of children disabled by age five report worse health than mothers of nondisabled children; however, this pattern is only evident among lower SES mothers and disappears for higher SES mothers. Contextualizing the findings within the systemic ableism literature highlights how—instead of portraying disabled children as burdens on their families—scholars and policymakers should focus on how ableism and poverty burden disabled people and their families in ways that pattern health risks.

Despite the growing call to integrate disability studies into sociological research on health and inequality (Mauldin and Brown 2021; Mladenov and Dimitrova 2022; Pettinicchio, Maroto, and Brooks 2022; Shandra 2018; Thomas 2004a, 2004b, 2007, 2022), sociological research often overlooks disability and ableism (Goodley and Runswick-Cole 2021; Green and Barnartt 2017; Shuttleworth and Meekosha 2013). Studies that do consider disability frequently reflect an assumption that disability is inherently negative or burdensome (Campbell 2009; Chennat 2019; Garland-Thomson 2013; Stoll and Egner 2021). An interdisciplinary body of literature examining the impacts of disabled children on their families, especially their mothers, widely participates in this narrative (Busch and Barry 2007; Carlson and Miller 2017). For example, studies document that mothers of disabled children report worse physical and mental health outcomes than mothers of nondisabled children (Ha et al. 2008; Olsson and Hwang 2001; Seltzer et al. 2009; Shivers and Resor 2020; Smith and Grzywacz 2014), although patterns vary across disability type (Seltzer et al. 2001; Sloan et al. 2020).

Grounded in the assumption that disability is negative or burdensome, some studies attempt to pinpoint specific attributes of the child’s disability that may be responsible for the worsened health of parents raising disabled children (Baker, Blacher, and Olsson 2005; Wang et al. 2004) or look to parents’ personal qualities to explain why some parents of disabled children fare better than others (Minnes, Perry, and Weiss 2015; Trute et al. 2010). Research that focuses on how some families learn to display resilience, cope, or adapt to raising a disabled child (Hall et al. 2012; Minnes et al. 2015; Murphy et al. 2006; Seltzer et al. 2004; Trute et al. 2010), while it seemingly reflects a more positive outlook, ultimately reflects the assumption that disabled children are a problem for parents and families to manage. Work describing the positive impacts of raising a disabled child in addition to the negative nonetheless reflects ableism in the sense that it implies disability is something negative that families must withstand or mitigate by focusing on the positive (Nurullah 2013; Trute et al. 2010; Trute, Hiebert-Murphy, and Levine 2007). Even studies that consider social factors can perpetuate ableist rhetoric by taking the point of view that greater financial resources can reduce the so-called burden of raising a disabled child (Kuhlthau et al. 2005).

Applying a critical disability studies perspective to the empirical work examining the relationship between children’s disability and maternal health highlights that the child’s disability *itself* does not negatively affect maternal health; rather, raising a disabled child in the context of social and structural environments that fail to meet the needs of disabled children and their families produces worse maternal health (Emerson et al. 2006; Lalvani and Polvere 2013; McLaughlin 2012; Olsson and Hwang 2008). As mothers raising disabled children help to meet their children’s access needs, they often encounter tremendous financial and bureaucratic challenges, reflecting the systemic privileging of nondisabled people (Baker and Burton 2018; Carey 2015; Resch et al. 2010). Families raising disabled children face substantial risk of poverty, material hardship, and lower economic well-being (Grech 2016; Kuhlthau et al. 2005; Parish et al. 2004). Over time, these financial and bureaucratic challenges may erode health. Thus, family socioeconomic status (SES) may be especially important in shaping the link between early childhood disability and maternal health.

Using data from the Future of Families and Child Wellbeing Study, this study uses multilevel modeling to examine the longitudinal relationship between early childhood disability—defined here as disability onset by age five—and maternal self-rated health among mothers living in large cities in the United States, paying particular attention to how family SES modifies this relationship. Scholars identify the first five years of motherhood as an important period for maternal well-being, work decisions, and adaptation to parenthood (Asmussen et al. 2016; Pontoppidan et al. 2022). Thus, mothers whose children are disabled prior to entering the school system may differ from mothers whose children are identified as disabled later. For example, mothers whose children are disabled during early childhood may be particularly susceptible to financial strain because they experience increased disability-related expenses (Morris et al. 2022) for longer periods of time. Decisions regarding childcare and labor market participation among mothers whose children are disabled in early childhood could impact their health and career trajectories over time. By focusing on early childhood disability, the disabilities represented in this study include primarily long-term disabilities that are more recognizable during early childhood.

In line with previous research, findings indicate that, on average, mothers of children who were disabled by age five report worse self-rated health than mothers of nondisabled children. However, results show that this relationship depends on family SES; lower SES mothers display the link between early childhood disability and maternal health, but higher SES mothers do not. These findings challenge the assumption that disability is inherently burdensome, revealing instead that it is ableism and poverty that burden disabled children and their families. Contextualizing this study within the sociological literature on ableism highlights the need for greater focus on how systems and policies have failed to support the basic needs of disabled children and their families in ways that pattern health risks, particularly in families with fewer socioeconomic resources.

## Background

### Understanding Disability

There are several approaches to conceptualizing disability in the social science literature, including the medical model, social model, a social relational understanding, and other critical disability studies perspectives. The medical model of disability suggests that disability is a biological impairment located exclusively in individual bodies, whereas the social model frames disability as the result of barriers in physical and social environments (Grue 2016; Oliver 1983; Thomas 2007). The medical model of disability is problematic because it ignores structure and perpetuates ableism. While the social model illuminates how structural forces shape disabled people’s lives, it fails to capture all dimensions of the lived experience of disabled people (Thomas 2004b). The social relational understanding of disability, which does not view the medical and social models as mutually exclusive, contends that disability arises when activity restrictions are socially imposed on people with impairments, resulting in the systematic exclusion of disabled people (Thomas 2004b). This perspective frames disability as a form of social oppression that occurs through relational encounters at the micro, meso, and macro levels, including family relationships, community interactions, and institutional encounters with health care, education, or welfare systems (Thomas 2004a, 2004b). A social relational perspective reflects an understanding that the social meaning of disability is reinforced in structural contexts and mediated through interpersonal dynamics, including family relationships, in ways that can compromise relationships and health (Brown and Ciciurkaite 2021; Carr, Cornman, and Freedman 2017; Friedman and Owen 2017).

Perspectives from critical disability studies also inform a more holistic conceptualization of disability that considers medical, social, and political aspects of disability. For example, critical disability theory conceptualizes disability as the interrelationship between impairment, response to impairment, and social and structural environments (Hosking 2008). Critical disability scholars understand disability as a matter of power distribution favoring nondisabled people (Lalvani and Polvere 2013; Schalk 2017; Thomas 2004b). Such power imbalances and the systematic oppression of disabled people and their families create the disadvantage families raising disabled children experience (Lalvani and Polvere 2013). Along with a social relational understanding of disability, a critical disability studies perspective illuminates the source of the disadvantage that mothers of disabled children experience. The child’s disability itself does not drive adverse maternal health outcomes; rather, social and structural factors interact with disability to produce disadvantage.

### Children’s Disability and Parental Health

A robust body of research, drawing mostly on a medical model of disability, finds a strong and persistent association between children’s disability and parental health. Compared with parents of nondisabled children, parents of disabled children experience higher rates of depression and anxiety (Hoyle, Laditka, and Laditka 2021; Olsson and Hwang 2001; Smith and Grzywacz 2014), lower self-rated and functional health (Burton, Lethbridge, and Phipps 2008; Murphy et al. 2006; Shivers and Resor 2020; Smith and Grzywacz 2014), more chronic health conditions (Ha et al. 2008; Raina et al. 2005), and higher stress (Carlson and Miller 2017; Miodrag et al. 2015; Miodrag and Hodapp 2010; Raina et al. 2005; Seltzer et al. 2009). Studies focused on early childhood find that mothers of young disabled children report higher child-related stress (Warfield et al. 1999) and lower family quality of life (Wang et al. 2004) than mothers of nondisabled children. These patterns differ by parental age, such that younger parents raising disabled children experience more adverse physical and mental health outcomes than older parents raising disabled children (Ha et al. 2008). Parental well-being also depends on the timing of disability onset (Seltzer et al. 2001, 2004). For example, parents of children whose disabilities began early in the life course must accommodate their family routines, care responsibilities, and labor market decisions early on, such that by midlife their well-being does not differ from parents of nondisabled children (Seltzer et al. 2001, 2004). By contrast, parents whose children develop disabilities later on during adolescence or young adulthood experience worse physical and emotional well-being than other parents at midlife (Seltzer et al. 2004).

Historically, much of the literature examining patterns of poor health among parents raising disabled children has focused on characteristics or behaviors of the child, ignoring the structural characteristics in which those elements exist. For example, some research has attributed the higher stress and worse health among parents of disabled children to children’s behavior problems (Baker et al. 2005; Miodrag and Hodapp 2010) or disability severity (Raina et al. 2004; Wang et al. 2004). Studies also compare maternal well-being across disability types, finding that mothers of children with mental health conditions and developmental disabilities, particularly autism, report higher stress and lower well-being than parents of children with other or no disabilities (Busch and Barry 2007; Seltzer et al. 2004; Sloan et al. 2020). Focusing on characteristics of the child’s disability reflects a tendency to frame disability as a burden on parents and society rather than focusing on the structural burdens that society places on disabled children and their families.

Likewise, studies examining parents’ coping strategies or resilience largely ignore structural factors and reflect an assumption that raising a disabled child has a negative impact on parents. Studies report that parents of disabled children fare better if they employ accommodative coping strategies (Minnes et al. 2015; Murphy et al. 2006; Seltzer et al. 2004; Trute et al. 2010), focus on the good aspects of raising a disabled child (Nurullah 2013; Trute et al. 2007, 2010), or display qualities such as optimism (Baker et al. 2005) or positive self-esteem (Trute et al. 2007). Such studies reflect the assumption that disability is inherently negative, for example, by discussing how parents find “hope in the midst of despair” (Nurullah 2013:23) or display resilience as they endure the “burden” of raising a disabled child (Hall et al. 2012; Murphy et al. 2006). These studies also place the responsibility on parents to cope or display resilience rather than addressing the structural factors underlying parents’ adverse health outcomes.

### Ableism and Stigma

Ableism is a system that values certain abilities, bodies, and minds over others, characterizing the experience of disability as inherently negative (Campbell 2009; Chennat 2019; Schalk 2017). It produces sociopolitical structures that uphold and value these abilities, resulting in the dehumanization and exploitation of disabled people (Chennat 2019; Nario-Redmond 2019). As such, disability implies stigma, marginalization, and notions of inferiority (Garland-Thomson 2013; Grue 2016). Stigma operates through interpersonal and structural pathways that heighten stress and social disadvantage, ultimately serving as a fundamental cause of population health inequalities (Hatzenbuehler, Phelan, and Link 2013). Although this study cannot directly measure ableism or stigma, these factors play a significant role in the variables it does measure—the socioeconomic resources and health of mothers raising disabled children.

Recent work highlights how disability and ableism are axes of inequality that shape health and well-being (Mauldin and Brown 2021; Shandra 2018). The stigma, interpersonal mistreatment, and institutional discrimination disabled people experience can take a toll on health (Brown 2017; Brown and Ciciurkaite 2021; Namkung and Carr 2019). Among parents of disabled individuals, stigma pertaining to embarrassment, shame, and daily discrimination is linked to worse self-rated health and more chronic conditions (Song, Mailick, and Greenberg 2018).

Mothers raising disabled children are marginalized by direct and vicarious stigma (Francis 2012; Green et al. 2005; Niedbalski 2022), which are mediated through interpersonal encounters. For example, people sometimes blame mothers for causing their child’s disability, label them as a bad parent, or judge them for their child’s behaviors (Francis 2012; Nurullah 2013). Mothers also navigate ableism, stereotypes, and stigma during encounters with health care, education, and social service environments (Green et al. 2005; Niedbalski 2022; Nurullah 2013). Disabled children and their families encounter barriers such as limited access to information and services and school and community exclusion (Resch et al. 2010). The bureaucratic challenges and structural hostility that parents of disabled children experience when navigating health care, education, and welfare systems make it difficult for families to obtain needed services and resources for their children (Baker and Burton 2018; Carey 2015; Hogan 2012; Thomas 2021). Families with limited financial resources may find these barriers in the physical, social, and service environments particularly challenging (Goodley and Runswick-Cole 2010).

### The Role of SES

Ableist structures that prioritize nondisabled people have largely placed the financial responsibility of medical care, therapies, equipment, transportation, education, and other accommodations for disabled children on individual families (Lukemeyer, Meyers, and Smeeding 2000; Vonneilich, Lüdecke, and Kofahl 2016), making the availability of socioeconomic resources salient for families with disabled children. Studies of the relationship between children’s disability and maternal health that address family SES suggest that it mediates this relationship (Sloper and Turner 1993; Vonneilich et al. 2016). Scholars have explored the socioeconomic implications of raising a disabled child, highlighting that financial strain is common among families raising disabled children (Galbraith et al. 2005; Kuhlthau et al. 2005; Parish et al. 2004) due to the extra costs associated with disability (Drew 2015; Lukemeyer et al. 2000; Morris et al. 2022). Relative to families without disabled children, families with disabled children have fewer socioeconomic resources (Shandra et al. 2012) and face greater risk of poverty and material hardship (Drew 2015; Grech 2016; Green 2007; Park, Turnbull, and Turnbull 2002). Disabled children disproportionately reside in low-income and single-mother households (Cohen and Petrescu-Prahova 2006), where the risk of material hardship is especially high (Parish et al. 2008).

Among families raising disabled children, partnered and single mothers alike experience high risk of income reduction. While paternal involvement is important for supporting the care needs of disabled children (Shandra, Hogan, and Spearin 2008), entrenched gender expectations mean that mothers of disabled children usually take on a disproportionate amount of care work (Blum 2015; Carey 2009; Leiter et al. 2004). Many mothers reduce work hours or leave the formal labor market to provide care for their disabled child (Blum 2015; Busch and Barry 2007; Carey 2009; Leiter et al. 2004; Parish et al. 2012; Shearn and Todd 2000). Mothers of children with developmental disabilities report lower employment rates at midlife (Seltzer et al. 2001) and lower income and savings over time (Parish et al. 2004) than their counterparts.

Among parents of disabled children, poverty is associated with lower health and well-being (Park et al. 2002). The adverse health outcomes among mothers of disabled children has been attributed, in part, to the increased risks of socioeconomic disadvantage among families raising disabled children (Emerson et al. 2006; Olsson and Hwang 2008). While studies document that children’s disability relates to maternal health through its influence on family SES, scholars have overlooked whether family SES also moderates the relationship between children’s disability and maternal health; thus, it is unknown whether the significance and magnitude of this relationship varies across different levels of family SES.

Whereas limited socioeconomic resources may increase health risks among mothers raising disabled children, greater socioeconomic resources may allow mothers to meet their disabled children’s needs in ways that mitigate health risks (Green 2007; Hogan 2012). Mothers of disabled children with greater family income report higher family quality of life (Wang et al. 2004). Higher SES families are better equipped to balance work and family life by outsourcing housework or controlling their work schedules (Nomaguchi and Milkie 2017), which may be especially important for parents caring for disabled children and navigating the additional time and costs associated with disability (Green 2007; Lukemeyer et al. 2000; Morris et al. 2022). Furthermore, higher SES families can mobilize their greater social, cultural, and economic capital to navigate financial and bureaucratic challenges in health care and education systems (Hogan 2012) and to garner necessary resources for caring for their disabled child (Green 2007). The literature examining the socioeconomic implications of raising a disabled child suggest that household SES may be a salient contextual factor linking early childhood disability and maternal health.

### Research Aims

While prior research identifies family SES as a mechanism mediating the relationship between children’s disability and maternal health, the potential *moderating* role of family SES remains unknown. Another open question is what maternal health trajectories look like for mothers of children who were disabled during early childhood. To address these gaps, I answer the following research question: How does family SES modify the relationship between early childhood disability and maternal self-rated health?

Merging insights from disability studies and medical sociology, this study aims to explore whether and how the magnitude of the association between early childhood disability and maternal health varies across household income-to-poverty levels among families living in large urban areas in the United States, comparing trajectories for mothers of children who were disabled during early childhood and mothers of nondisabled children. I hypothesize that among lower SES families, mothers of children who were disabled by age five will have lower self-rated health over time than mothers of nondisabled children. By contrast, among higher SES families, I anticipate that mothers of children who were disabled by age five and mothers of nondisabled children will display similar health trajectories. By examining how the relationship between early childhood disability and maternal health depends on SES, this study improves understanding of the role of social and structural contexts in shaping the links between early childhood disability and maternal health.

## Data and Methods

### Data and Analytic Sample

This study drew on data from the Future of Families and Child Wellbeing Study, a longitudinal study of 4,898 families with a child born between 1998 and 2000 in large cities geographically dispersed throughout the contiguous United States. Researchers gathered data in six waves from the birth of the child through age 15 (Years 0, 1, 3, 5, 9, and 15). They oversampled mothers who were not married at the time of childbirth and weighted the data to make it representative of all births between 1998 and 2000 in U.S. cities with populations of 200,000 people or more. The longitudinal data contained measures of maternal health, children’s disability status, and household SES.

I restricted my sample to biological mothers who lived with the child at least some of the time and had valid outcome data. I excluded mothers of children whose disability was identified after age five so that the comparison groups include mothers of children who were disabled during early childhood and mothers of nondisabled children. Because the proportion of missingness per variable was less than 1% for each covariate, I dropped respondents with missing data. The analytic sample size included 5,575 person-year observations from 2,338 unique individuals, consisting of 1,784 mothers at Year 5, 1,958 mothers at Year 9, and 1,833 mothers at Year 15. The final sample consisted of 577 mothers whose children were disabled by age five and 1,761 mothers of nondisabled children.

### Measures

#### Maternal health

The dependent variable for this analysis was maternal self-rated health. Mothers were asked, “In general, how is your health?” Responses were coded such that 1 = poor health, 2 = fair health, 3 = good health, 4 = very good health, and 5 = excellent health. In preliminary analyses, I ran models estimating self-rated health as an ordinal outcome variable versus a continuous outcome variable. Results were substantively similar regardless of the operationalization of self-rated health. For ease of interpretation, I operationalized maternal health as a continuous measure in the analysis presented here.

#### Early childhood disability

The primary independent variable was early childhood disability, using age five as the cutoff for “early childhood.” Early childhood disability was a time-constant predictor where 1 = child was disabled by age five and 0 = nondisabled child. I categorized respondents as mothers of children who were disabled by age five if they reported that their child had cerebral palsy, Down syndrome, blindness, deafness, problems with limbs, other physical disability, autism, ADHD, intellectual disability or developmental delays, seizures/epilepsy, or speech or language problems at Years 1, 3, or 5. By focusing on early childhood disability, this study primarily included children with lifelong or early onset physical disabilities and developmental disabilities that were more readily apparent at a young age.

#### Family SES: household income-to-poverty level

I used a time-varying categorical measure of household income relative to the federal poverty level (FPL) to measure family SES. The U.S. Census Bureau’s official poverty measure, an absolute poverty measure based on household size and composition, supplied the measurement for categorizing household income-to-poverty level categories. The 100% FPL threshold undercounts families experiencing economic hardship generally and undercounts material deprivation among families with a disabled member particularly (Morris et al. 2022). Therefore, I took the approach of using less than 200% FPL as the threshold to capture lower SES families (Galbraith et al. 2005). In this analysis, household income-to-poverty level categories included 0% to 199% FPL, 200% to 299% FPL, and 300%+ FPL. The reference group was lower SES households (0%–199% FPL).

#### Covariates

I included several maternal covariates to account for factors that may correlate with maternal health trajectories and the likelihood of having a disabled child. The covariates included measures of educational attainment (1 = college degree, 0 = no college degree), age at time of childbirth (centered on the mean), baseline health (1 = poor or fair health, 0 = good or better health), and race-ethnicity (non-Hispanic Black, Hispanic/Latinx, non-Hispanic White [reference], non-Hispanic “other” race) at baseline. In the multilevel models, I used the interview year (child’s age) as the time variable. Centering this variable at Year 5 allowed me to examine trajectories of maternal self-rated health from the end of early childhood (Year 5) through adolescence (Year 15). I interpreted the initial status in maternal health as the mother’s self-rated health when the child was five years old, adjusting for other covariates.

In supplementary analyses, I explored other possible covariates, including marital or cohabitation status and household composition measures, such as how many children and adults lived in the household. I did not find any statistically significant associations between these variables and the outcome, and including these variables in the models did not substantively change the results. Thus, I excluded them in favor of a more parsimonious model with improved model fit.

### Analytic Approach

First, I calculated weighted descriptive statistics for the full sample and by early childhood disability, using *t* tests and χ^2^ tests to compare baseline sample characteristics for mothers of children who were disabled by age five and mothers of nondisabled children.

Next, I used multilevel linear mixed effects models to estimate maternal self-rated health trajectories from Year 5 to 15. These models included fixed effects parameters and variance components for the random effects. Multilevel models were appropriate for this longitudinal data analysis for several reasons. First, the data have multiple observations nested within individual mothers, and the models accounted for the correlation in error terms resulting from following the same mothers over time. Second, these models can still produce unbiased estimates with unbalanced data, so the mothers did not have to have the same number of observations. Third, the models allowed for including both time-varying and time-constant variables. Finally, by containing two levels, the models simultaneously considered within-person and between-person changes over time. Level 1 captured how the health of each mother changed over time (within-person changes). Level 2 captured how these changes varied across different mothers (between-person changes). Altogether, the models described how maternal health changed over time and whether these trajectories differed between mothers of children disabled by age five and mothers of nondisabled children.

I presented results from three stepwise multilevel models. For all models, Level 1 contained individual growth parameters, including the true initial status (*π_0i_*) and rate of change (*π_1i_*) plus the residual errors. For Model 1, Level 2 included early childhood disability, which affected both the initial status and rate of change but no other covariates. For Model 2, Level 2 included early childhood disability, household income-to-poverty, and the baseline covariates, all of which affected the initial status and rate of change. For Model 3, I added the interaction terms between early childhood disability and household income-to-poverty categories into Level 2, affecting the initial status. The interaction term between early childhood disability and household income-to-poverty assessed whether the association between early childhood disability and maternal health depended on family SES.

I assessed the goodness of fit of the stepwise models using Akaike information criterion (AIC) and deviance statistics. AIC determined the best-fitting model based on which model explained the greatest amount of variation in maternal health with the fewest number of independent variables. Deviance did not penalize for the number of independent variables in the model; rather, it assessed how well a model fit the data based on the difference of log likelihoods between the specified model and a hypothetical saturated model that perfectly fit the data. In both cases, the lower the statistic, the better the model fit is. The model comparison indicated that Model 3 best fit the data because it had the lowest AIC and deviance. Based on Model 3, I plotted the predicted trajectories of maternal self-rated health from Year 5 to 15 by early childhood disability and household income-to-poverty level. I specified the Level 1 and Level 2 submodels for Model 3 in the following:

*Level 1*:

(1) $$Y_{ij}=\pi_{0i}+\pi_{1i}(Year_{ij}-5)+\varepsilon_{ij}.$$

*Level 2*:

(2) $$\begin{array}{l} \pi_{0i}=\gamma_{00}+\gamma_{01}(Child\;Disability{)}_{i}+\gamma_{02}(Age{)}_{i}\\ \;\;\;\;+\gamma_{03}(College{)}_{i}+\gamma_{04}(Poor\;or\;Fair\;Health{)}_{i}\\ \;\;\;\;+\gamma_{05}(Black{)}_{i}+\gamma_{06}(Hispanic/Latinx{)}_{i}\\ \;\;\;\;+\gamma_{07}(Other\;Race{)}_{i}+\gamma_{08}(200\;to\;299\;FPL{)}_{ij}\\ \;\;\;\;+\gamma_{09}(300\;plus\;FPL{)}_{ij}\\ \;\;\;\;+\gamma_{010}(Child\;Disability{)}_{i}(200\;to\;299\;FPL{)}_{ij}\\ \;\;\;\;+\gamma_{011}(Child\;Disability{)}_{i}(300\;plus\;FPL{)}_{ij}+\zeta_{0i}. \end{array}$$

(3) $$\begin{array}{l} \pi_{1i}=\gamma_{10}+\gamma_{11}(Child\;Disability{)}_{i}\\ \;\;\;\;+\gamma_{12}(Age{)}_{i}+\gamma_{13}(College{)}_{i}\\ \;\;\;\;+\;\gamma_{14}(Poor\;or\;Fair\;Health{)}_{i}+\gamma_{15}(Black{)}_{i}\\ \;\;\;\;+\gamma_{16}(Hispanic/Latinx{)}_{i}+\gamma_{17}(Other\;Race{)}_{i}\\ \;\;\;\;+\gamma_{18}(200\;to\;299\;FPL{)}_{ij}\\ \;\;\;\;+\gamma_{19}(300\;plus\;FPL{)}_{ij}+\zeta_{1i}. \end{array}$$

## Results

### Descriptive Analysis

Table 1 shows weighted descriptive statistics among the full sample and stratified by mothers of children disabled by age five versus mothers of nondisabled children. Approximately 28% of mothers have a child who was disabled by age five. In the full sample, nearly a quarter of mothers had a college degree or higher, and only 6% reported poor or fair health at baseline. Roughly 26% of mothers are non-Hispanic Black, 31% are Hispanic/Latinx, 36% are non-Hispanic White, and 7% identify with another race. Around half of the sample fell below 200% FPL at baseline.

**Table 1.** Weighted Sample Characteristics.

Variables	Full Sample (*N* = 2,338)	Mothers of Children Disabled by Age 5(*n* = 577)	Mothers of Nondisabled Children(*n* = 1,761)	*p* Value
Self-rated health (mean)	3.64	3.41	3.74	.0000***
Child disabled by age 5 (%)	28			
College degree or more (%)^a^	24	20	26	.0633
Age at childbirth (mean)^a^	27.09	26.86	27.15	.4081
Poor or fair health (%)^a^	6	11	5	.0001***
Race-ethnicity (%)^a^				.2486
Black, non-Hispanic	26	25	26	
Hispanic/Latinx	31	33	30	
Other race, non-Hispanic	7	5	8	
White, non-Hispanic	36	37	36	
HH income-to-poverty (%)				.0397*
0%–199% FPL	52	57	50	
200%–299% FPL	15	15	15	
300%+ FPL	33	28	35	

*Source*: The Future of Families and Child Wellbeing Study (1998–2017).

*Note*: I used two-tailed *t* tests for continuous variables and χ^2^ tests of independence for categorical variables to compare the outcome and covariates between mothers of children disabled by age five and mothers of nondisabled children. HH = household; FPL = federal poverty level.

a Measured at baseline (year child was born).

* *p* < .05, ***p < .001.

Mothers of children disabled by age five and mothers of nondisabled children exhibited statistically significant differences in health and family SES. Compared with mothers of nondisabled children, mothers of children who were disabled by age five were more likely to report poor or fair baseline health and have worse self-rated health across Years 5 to 15. Examining the distribution of household income-to-poverty by early childhood disability reveals an unequal distribution of socioeconomic resources, with mothers of nondisabled children reporting greater household income relative to the FPL compared with mothers of children disabled by age five. Baseline education, age, and race-ethnicity did not significantly differ between mothers of children disabled by age five and mothers of nondisabled children.

### Multilevel Models

Table 2 displays the results for the multilevel linear mixed effects models estimating maternal self-rated health from Year 5 to 15. The primary variable of interest, early childhood disability, remains negative and statistically significant across all models, indicating that on average, mothers of children disabled by age five have worse self-rated health than mothers of nondisabled children at year five. In Model 3, early childhood disability has a small but statistically significant rate of change parameter, showing that the health gap between mothers of children disabled by age five and mothers of nondisabled children converged slightly over time.

**Table 2.** Multilevel Linear Mixed Effects Models Estimating Maternal Self-Rated Health.

		Model 1	Model 2	Model 3
		*b* (SE)	*b* (SE)	*b* (SE)
Fixed effects				
Initial status, π_0*i*_	Intercept	3.743*** (.026)	3.973*** (.114)	3.991*** (.114)
	Child disabled by age 5	–.247*** (.051)	–.185*** (.050)	–.266*** (.054)
	Age at childbirth (y0)		–.017*** (.004)	–.016*** (.004)
	College degree+ (y0)		.410*** (.076)	.416*** (.076)
	Poor or fair health (y0)		–.718*** (.089)	–.714*** (.089)
	Race-ethnicity (y0)			
	Black, non-Hispanic		.179** (.059)	.184** (.060)
	Hispanic/Latinx		.072 (.062)	.076 (.062)
	Other race, non-Hispanic		–.133 (.117)	–.113 (.117)
	White, non-Hispanic			
	HH income-to-poverty			
	0%–199% FPL			
	200%–299% FPL		.115* (.050)	.057 (.055)
	300%+ FPL		.324*** (.051)	.243*** (.055)
	Disability × Poverty			
	Disability × 0%–199% FPL			
	Disability × 200%–299% FPL			.183* (.078)
	Disability × 300%+ FPL			.309*** (.077)
Rate of change, π_1*i*_	Child’s age	.018*** (.004)	–.038* (.016)	–.038* (.016)
	Child disabled by age 5	.003 (.007)	.004 (.007)	.001* (.001)
	Age at childbirth (y0)		.001* (.001)	.001* (.001)
	College degree+ (y0)		–.006 (.010)	–.006 (.010)
	Poor or fair health (y0)		–.001 (.013)	–.002 (.013)
	Race-ethnicity (y0)			
	Black, non-Hispanic		–.020* (.009)	–.020* (.009)
	Hispanic/Latinx		–.011 (.009)	–.011 (.009)
	Other race, non-Hispanic		.019 (.015)	.019 (.015)
	White, non-Hispanic			
	HH income-to-poverty			
	0%–199% FPL			
	200%–299% FPL		–.010 (.009)	–.009 (.009)
	300%+ FPL		–.008 (.008)	–.008 (.008)
Random effects				
Level 1 variance	Within-person (residual)	128.863	127.209	126.422
Level 2 variance	In initial status	.636	.565	.567
	In rate of change	.006	.006	.006
	Covariance	–.028	–.029	–.030
Goodness of fit				
	AIC	17,110.3	16,972.51	16,965.98
	Deviance	17,065.95	16,791.41	16,774.2

*Source*: The Future of Families and Child Wellbeing Study (1998–2017).

*Note*: *N* = 5,575 person-years, 2,338 unique individuals. The confidence intervals for the interaction terms between early childhood disability and household income to poverty are (.182, .183) for the Disability × 200%–299% FPL coefficient and (.308, .309) for the Disability × 300%+ FPL coefficient. HH = household; FPL = federal poverty level; AIC = Akaike information criterion.

* *p* < .05, **p < .01 ***p < .001 (two-tailed *t* tests).

The estimated income-to-poverty parameters indicate that higher SES mothers have better self-rated health at year five than lower SES mothers. The associations between income-to-poverty and maternal self-rated health are constant over time. The interaction terms between early childhood disability and household income-to-poverty in Model 3 reveal that net of all other variables, the association between early childhood disability and maternal health varies by SES. After accounting for other variables, I find that mothers of children disabled by age five report worse self-rated health than mothers of nondisabled children among low-income households, but self-rated health does not differ between mothers of children disabled by age five and mothers of nondisabled children among higher-income households.

**Figure 1.**
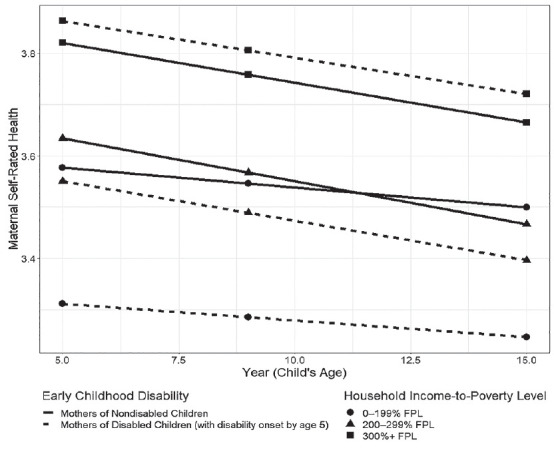
Predicted Maternal Self-Rated Health Trajectories by Early Childhood Disability and Household Income-to-Poverty Level. *Source*: The Future of Families and Child Wellbeing Study (1998–2017). *Note*: Predictions are based on reference group in Model 3. I excluded confidence intervals for clarity of data visualization. Among 0%–199% FPL households, the gap in self-rated health between mothers of children disabled by age five and mothers of nondisabled children is statistically significant, with mothers of children disabled by age five reporting worse health than mothers of nondisabled children. Among 200%–299% and 300%+ FPL households, the gap in self-rated health between mothers of children disabled by age five and mothers of nondisabled children is not statistically different. FPL = federal poverty level.

Figure 1 visually depicts this result, showing that the predicted trajectories of maternal self-rated health by early childhood disability are not equivalent across household SES. Maternal health reflects a socioeconomic gradient in maternal health; regardless of children’s disability, mothers with household incomes 300% FPL or above have the highest predicted self-rated health. Among mothers living in lower SES households (0% -199% FPL), mothers of nondisabled children experience higher self-rated health at all time points compared with mothers of children disabled by age five, adjusting for baseline covariates.

However, a different pattern emerges among higher SES mothers. The self-rated health disparity between mothers of children disabled by age five and mothers of nondisabled children disappears among mothers with household incomes 200% to 299% FPL and 300% FPL or above. Moreover, income-stratified models reveal no significant difference in self-rated health between mothers of children disabled by age five and mothers of nondisabled children, accounting for baseline covariates. While previous scholarship suggests that mothers of disabled children report worse health than mothers of nondisabled children, my findings suggest that the association between early childhood disability and maternal health is only significant among mothers in lower SES households.

## Discussion

A large body of research shows that compared with mothers of nondisabled children, mothers of disabled children report worse physical and mental health. This study expands current knowledge by using multilevel modeling and longitudinal data from the Future of Families and Child Wellbeing Study to explore how household SES modifies the relationship between early childhood disability and maternal health among a sample of families living in large U.S. cities. Findings identify persistent health inequalities between mothers of children who were disabled by age five and mothers of nondisabled children among lower SES families, with mothers of nondisabled children reporting better self-rated health at all time points. However, higher SES families do not display this pattern; early childhood disability does not predict the health of mothers with household incomes 200% FPL or above.

These findings support my hypothesis that the relationship between early childhood disability and maternal health depends on household SES. Although this study cannot directly measure ableism, its findings challenge the ableist rhetoric that disability is inherently negative or burdensome. Situating these findings within the sociological and critical disability studies literature on ableism helps to explain why the relationship between early childhood disability and maternal health varies across family SES. By assigning value to certain bodies and minds over others (Campbell 2009; Chennat 2019; Schalk 2017), ableism creates and perpetuates disability stigma and structural discrimination (Campbell 2009; Nario-Redmond 2019), marginalizing both disabled children and their mothers (Francis 2012; Green et al. 2005; Niedbalski 2022). Mothers raising disabled children must navigate ableist structures and relational encounters that require greater time and money—resources that vary widely depending on household SES (Green 2007; Thomas 2022).

Interlocking systems of ableism and capitalism have led to the commodification of disability, which renders meeting a disabled person’s needs expensive and creates systems that profit from services designed to meet those needs (Pedlar and Hutchison 2000). For example, vehicle adaptations, home modifications, mobility aids, assistive technologies, and medical, educational, and therapeutic services come with steep price tags (Lukemeyer et al. 2000; Vonneilich et al. 2016). In the United States, the extra costs associated with disability largely fall on individual families due to inadequate government support (Drew 2015; Lukemeyer et al. 2000; Morris et al. 2022). Higher SES mothers may be able to avoid some of the structural ableism, stigma, marginalization, and blame that, as a social relational understanding of disability indicates (Thomas 2004a, 2004b), can undermine relational interactions in ways that impact health (Brown 2017; Brown and Ciciurkaite 2021; Carr et al. 2017; Friedman and Owen 2017; Mauldin and Brown 2021; Namkung and Carr 2019), including among mothers raising disabled children (Francis 2012; Green 2003; Green et al. 2005; Niedbalski 2022; Nurullah 2013; Thomas 2021). Unrelenting disability-related costs and institutional barriers rooted in ableism may be particularly challenging for mothers of disabled children with limited socioeconomic resources, explaining the health disparity that this study observes among lower SES mothers but not higher SES mothers.

This study has several limitations. First, the results cannot be generalized beyond large U.S. cities. The barriers mothers navigate in rural and suburban areas and their alignment with SES may look different than in large, urban areas. For example, higher SES may not make accessing care and disability-related services much more accessible in less densely populated areas. Future research is needed to assess the extent to which the results of this study apply in other settings.

The official poverty measure also imposes limitations because it undercounts families facing economic hardship. Future studies could use more comprehensive measures of poverty, such as relative poverty or the Supplemental Poverty Measure, which may improve the reliability and validity of poverty estimates. Additionally, poverty is just one domain of SES. Thus, future research could consider other aspects of SES, including educational attainment, wealth, occupation, material hardship, and flexible resources apart from money.

The data set also imposes limitations. Future research should address the limitation created by the fact that the data do not include measures of systemic ableism. Future studies could incorporate measures of ableism, such as perceived stigma, day-to-day discrimination, microaggressions, and institutional discrimination (Brown 2017; Friedman and Awsumb 2019; Kattari 2019; Namkung and Carr 2019), and measures of barriers within physical and social environments that impact disabled people and their families (Latham-Mintus and Cordon 2021). Additionally, the Future of Families and Child Wellbeing Study did not ask the same set of disability questions at every wave of data. While comparing mothers of children who were disabled by age five and mothers of nondisabled children aligned with theoretical understandings of the importance of early childhood and early motherhood, the focus on early childhood disability limited the types of disabilities captured in this study. Children with intellectual and learning disabilities experience especially severe marginalization (Carey 2009), but these disability types are underrepresented in this study because they are often not recognized until school age. Future research should employ time-varying measures of disability and include mothers of children whose disabilities were not recognized until later on. Comparing disability types was also precluded due to small sample sizes. Future scholarship could assess heterogeneity across disability types because the costs and stigma associated with raising a disabled child vary by disability type. Data limitations point to a wider need for improved measures of disability in longitudinal survey data, including measures rooted in a social relational understanding of disability.

Despite these limitations, this study has important implications for policy and scholarly efforts aimed at alleviating maternal health disparities. If society continues to focus on individual-level intervention strategies, such as teaching parents resiliency and accommodative coping strategies or alleviating child behavior problems, health disparities between mothers of children with and without disabilities will likely persist. Scholars and policymakers should focus their attention on how policies have failed to support the basic needs of disabled children and their families, particularly families with fewer socioeconomic resources.

To reduce financial strain and improve maternal health, policies need to minimize the extra costs associated with disability and expand systems of care that are rooted in interdependence. Among families raising disabled children, those who live in states with higher Medicaid coverage report less financial hardship (Parish, Shattuck, and Rose 2009). Thus, states should increase Medicaid coverage to reduce out-of-pocket expenses for families raising disabled children, which would particularly benefit lower SES families raising disabled children (Parish et al. 2009, 2012). State and federal governments need to provide comprehensive insurance coverage, cash supports, and home- and community-based services for disabled children and their families (Busch and Barry 2007; Kuhlthau et al. 2005). Policies are also needed to provide care support and help mothers balance the demands of work and family. Government childcare subsidies and flexible work policies would provide mothers with the time and resources needed to care for their children (Nomaguchi and Milkie 2017; Shearn and Todd 2000). Policy and scholarly efforts to advance health equity for mothers of disabled children must align with the principles of disability justice (Sins Invalid 2016) and focus on dismantling systemic ableism or else risk perpetuating the marginalization of disabled children and their families.

## Conclusion

Building on the movement to integrate disability studies and medical sociology (Mauldin and Brown 2021; Mladenov and Dimitrova 2022; Thomas 2004a, 2007, 2022), this study contributes empirical evidence that the relationship between early childhood disability and maternal health depends on family SES among families in large U.S. cities. Findings from multilevel models indicate that while mothers of children who were disabled by age five reported lower self-rated health than mothers of nondisabled children among lower SES households, this health gap disappeared among more-resourced mothers. Contextualizing these findings within the literature on systemic ableism (Campbell 2009; Hosking 2008; Lalvani and Polvere 2013; Stoll and Egner 2021; Thomas 2021) challenges the pervasive assumption that disability is inherently negative or burdensome. Social and structural contexts rooted in ableism that marginalize disabled children and their families—not the child’s disability—drive the adverse health outcomes that lower SES mothers raising disabled children experience. This study’s finding that a family’s access to resources and position within social hierarchies determines maternal health reveals this distinction and demonstrates that disability is not a burden. Rather than perpetuating the ableist rhetoric that disabled children are burdens on their families and communities, future research and policy efforts must focus on how ableism and poverty burden disabled people and their families.
